# Differential microbial assembly processes and co‐occurrence networks in the soil‐root continuum along an environmental gradient

**DOI:** 10.1002/imt2.18

**Published:** 2022-04-05

**Authors:** Yangquanwei Zhong, Patrick O. Sorensen, Guangyu Zhu, Xiaoyu Jia, Jin Liu, Zhouping Shangguan, Ruiwu Wang, Weiming Yan

**Affiliations:** ^1^ School of Ecology and Environment Northwestern Polytechnical University Xi'an P.R. China; ^2^ Earth and Environmental Sciences Lawrence Berkeley National Laboratory Berkeley California USA; ^3^ College of Environment and Ecology Chongqing University Chongqing P.R. China; ^4^ State Key Laboratory of Soil Erosion and Dryland Farming on the Loess Plateau Northwest A&F University Yangling P.R. China

**Keywords:** assembly process, community dissimilarity, environmental gradient, network, root‐associated microbiomes, soil microbe

## Abstract

Microorganisms of the soil‐root continuum play key roles in ecosystem function. The Loess Plateau is well known for its severe soil erosion and thick loess worldwide, where mean annual precipitation (MAP) and soil nutrients decrease from the southeast to the northwest. However, the relative influence of environmental factors on the microbial community in four microhabitats (bulk soil, rhizosphere, rhizoplane, and endosphere) in the soil‐root continuum along the environmental gradient in the Loess Plateau remains unclear. In this study, we investigated 82 field sites from warm‐temperate to desert grasslands across the Loess Plateau, China, to assess the bacterial diversity, composition, community assembly, and co‐occurrence networks in the soil‐root continuum along an environmental gradient using bacterial 16S recombinant DNA amplicon sequencing. We discovered that the microhabitats explained the largest source of variations in the bacterial diversity and community composition in this region. Environmental factors (e.g., MAP, soil organic carbon, and pH) impacted the soil, rhizosphere, and rhizoplane bacterial communities, but their effects on the bacterial community decreased with increased proximity to roots from the soil to the rhizoplane, and the MAP enlarged the dissimilarity of microbial communities from the rhizosphere and rhizoplane to bulk soil. Additionally, stochastic assembly processes drove the endosphere communities, whereas the soil, rhizosphere, and rhizoplane communities were governed primarily by the variable selection of deterministic processes, which showed increased importance from warm‐temperate to desert grasslands. Moreover, the properties of the microbial networks in the rhizoplane community indicate more stable networks in desert grasslands, likely conferring the resistance of microbial communities in higher stress environments. Collectively, our results showed that the bacterial communities in the soil‐root continuum had different sensitivities and assembly mechanisms along an environmental gradient. These patterns are shaped simultaneously by the intertwined dimensions of proximity to roots and environmental stress change in the Loess Plateau.

## Highlights

- Changes in microbes in the soil‐root continuum along an environmental gradient were investigated.
- The soil, rhizosphere and rhizoplane communities were governed by deterministic processes.
- Environmental stress enlarged the dissimilarity of microbial communities in the soil‐root continuum.
- Root‐associated microbiomes did not destabilize at locations with higher environmental stress.

## Graphical abstract

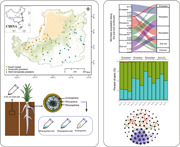

## INTRODUCTION

Soils harbor highly diverse microbial communities with numerous functions [1] and provide a seed bank for root‐associated microbiota that may contribute to plant growth, nutrient uptake, disease resistance, and stress tolerance [2]. Additionally, plants can selectively recruit potentially beneficial microbes through root exudates and deposits [3, 4]. The microbiota in the soil‐root continuum (bulk soil, rhizosphere, rhizoplane, and endosphere) are influenced directly/indirectly by biotic and abiotic factors, such as climatic factors, soil physical and chemical properties, and host plant traits [2, 5, 6, 7, 8, 9]. However, systematic studies considering the microbial community in four microhabitats along the soil‐root continuum across large geographic scales are still limited [10, 11].

At regional to global scales, precipitation and soil pH are critical drivers of soil microbial diversity and community structure [1, 12, 13, 14]. These climatic and edaphic factors may directly shape the assembly of microbiota in soil and contribute to the recruitment of microorganisms to the rhizosphere by plant roots [2, 7, 15]. However, the different niche and life history strategies of the microbiota in the soil‐root continuum may result in different responses of the microbial community to environmental factors. Certain studies have characterized the root‐associated microbiota using molecular‐based methods under different environmental conditions [2, 16, 17, 18, 19]. For example, aridity differentially influences microbial communities in the rhizosphere and endosphere [20]. Likewise, changes in altitude affect microbial community composition more in the rhizosphere than in bulk soil [21]. However, the changes in the microbial community in the soil‐root continuum along an environmental gradient remain poorly understood.

Microbial community assembly patterns are widely influenced by both deterministic and stochastic ecological processes [22, 23]. Deterministic processes involve nonrandom and niche‐based mechanisms [24], including environmental filtering and interspecific interactions (e.g., competition, facilitation, mutualisms, and predation). By contrast, stochastic processes mainly reflect random changes in the relative abundance of species—for example, random birth, death, and dispersal events [25, 26]. Microbial community assembly patterns in the soil‐root continuum are complicated and remain controversial because of the differential regulation mechanisms by root exudates [27]. Additionally, assembly processes are environmentally sensitive, and their relative proportion could reflect the community response to environmental change [28]. However, changes in community assembly patterns in the soil‐root continuum across spatially broad environmental gradients remain unknown, limiting our understanding of microbial community assembly patterns under ongoing climate change [27, 29].

The relationships among microbial taxa exert considerable influence over a range of host–microbe interactions, such as supporting plant growth, promoting disease resistance, and more generally enhancing the resilience and resistance of ecosystems to environmental disturbance [30, 31]. Such complicated ecological relationships can be represented in co‐occurrence networks [30, 32]. Changes in microbial networks might represent changes in interactions among coexisting organisms that could alter soil function or vulnerability to environmental disturbances [33], and networks with weak or negative interactions suggested increased stability and resistance to environmental disturbances [33, 34]. de Vries et al. [33] reported that drought promoted destabilizing properties in networks of soil bacteria that were associated with changes in vegetation composition and reductions in soil water availability. Shi et al. [35] investigated the networks of soil and rhizosphere microbes in a greenhouse microcosm experiment and found that networks of rhizosphere microbes were more complex than the networks in surrounding soils. However, the degree to which microbial co‐occurrence networks vary within and among soil and root‐associated microbiomes along environmental gradients in situ remains unclear.

To address these issues, we analyzed the bacterial communities in the soil‐root continuum surveyed at 82 field sites from warm‐temperate grasslands to desert grasslands of the semiarid and arid Loess Plateau, China, that is well known for its thick loess and severe soil erosion. The seasonal distribution of precipitation is uneven, as 60%–70% of the annual total precipitation occurs between June and September [36], making this region sensitive to environmental change [37]. Across our sampling sites in the Loess Plateau, the mean annual precipitation (MAP), mean annual temperature (MAT), and soil nutrients decreased from the southeast to northwest, resulting in distinct soil microbial communities [38]. Assessing bacterial diversity, community assembly and network associations in the soil‐root continuum along the environmental gradient (i.e., precipitation and temperature, edaphic properties, and plant traits) in this region could improve understanding of the microbial response to ongoing climate change. Based on this large‐scale data set, we hypothesized that microbial diversity and community composition would be considerably structured by niche differentiation in the soil‐root continuum and would differ along the environmental gradient. We also hypothesized that the deterministic bacterial community assembly process along the soil‐root continuum would increase with an increase in environmental stress (decreasing MAP and nutrient availability). Finally, we hypothesized that environmental stress would increase community dissimilarity along the soil‐root continuum and destabilize co‐occurrence networks.

## RESULTS

### Diversity and community composition of microbes in the soil‐root continuum along the environmental gradient

**Figure 1:**
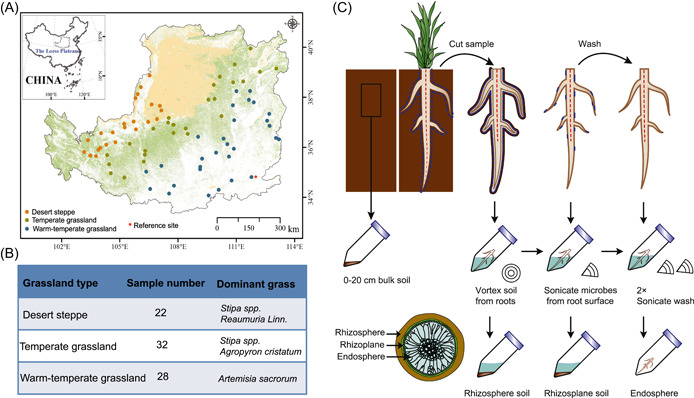
Sampling site locations and sampling methods. (A) The study sites on the Loess Plateau. The sites spanned a longitudinal range from 103°E to 112°E and a latitudinal range from 34°N to 40°N. (B) Sample numbers in each grassland type and dominant vegetation. (C) Methods of sampling and collection of the soil and root‐associated microbes

**Figure 2:**
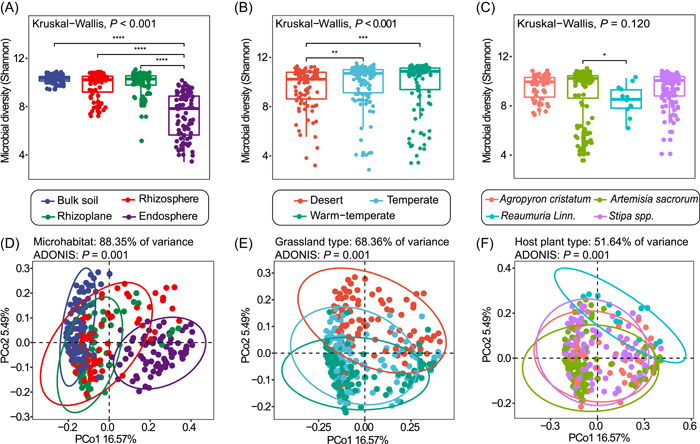
Soil and root‐associated bacterial community diversity and structure among different microhabitats and grassland types. (A–C) Shannon index of the microbiota in the soil‐ and root‐associated microbiome (A), different grassland types (B), and different host plant types (C). The horizontal bars within boxes represent medians. The tops and bottoms of boxes represent the 75th percentiles and 25th percentiles, respectively. The asterisk above the horizontal line represents a significant difference between the two groups, *p < 0.05, **p < 0.01, and ***p < 0.001. (D–F) Unconstrained PCoA with unweighted UniFrac distances that separated by microhabitats (D), grassland types (E), and host plant types (F) (*p* < 0.001, permutational multivariate analysis of variance [PERMANOVA] by Adonis). In the desert steppe, the major species are *Stipa* spp. and *Reaumuria Linn*.; temperate grasslands are dominated by *Stipa* spp. and *Agropyron cristatum*; warm temperate grasslands are dominated by *Artemisia sacrorum*. Percentages of variance above the panel indicate the results of the canonical analysis of principal coordinates (CAP) to better quantify the influence of these factors on the beta diversity. Ellipses cover 95% of the data for each group. The numbers of replicated samples in this figure are listed as follows: (A and D) soil (*n* = 82), rhizosphere (*n* = 82), rhizoplane (*n* = 82), and endosphere (*n* = 82); (B and E) desert (*n* = 88), temperate (*n* = 128), and warm‐temperate (*n* = 112). (C and F) *Agropyron cristatum* (*n* = 48), *Artemisia sacrorum* (*n* = 120), *Reaumuria Linn*. (*n* = 12), *Stipa* spp. (*n* = 112)

To investigate the microbes in the soil‐root continuum along an environmental gradient, 82 grasslands sites were selected in the Loess Plateau based on climate and vegetation characteristics (Figure 1). Based on the environmental factor changes across site, the environmental gradient across our study sites was driven mainly by differences in MAP, soil nutrient levels, and soil pH (Figure S1 and Table S1). We found that the microhabitat, grassland type, and host plant significantly affected bacterial diversity (Table S2). The microbial α‐diversity (Shannon index in our study) was the lowest in the endosphere (Figures 2A and S2A), and no significant differences were observed among the rhizoplane, rhizosphere, and bulk soil communities. The Shannon diversity in the desert grasslands was significantly lower than that in the temperate and warm‐temperate grasslands (Figures 2B and S2B). Additionally, the Shannon diversity varied among the different host plants (Figure 2C) but only showed differences in the endosphere and rhizosphere among the host plant types (Figure S2C).

The microhabitat, grassland type, and host plant also affected the bacterial community structure (ADONIS *p* < 0.01; Figures 2D–F and S3). PCoA based on the unweighted UniFrac distance showed more distinct separation than the weighted UniFrac distance among the different microhabitats, grassland types and host plants (Figures 2D–F, S3, and S4). Additionally, the CAP results revealed that the microhabitat significantly affected the composition of the microbiota (88.35% for unweighted UniFrac distance and 79.40% for weighted UniFrac distance; Figures 2D and S3A). The grassland types explained 68.36% and 47.46% of the variance in the bacterial community composition (Figures 2E and S3B), and the host plants explained 51.64% and 46.50% of the variance in the bacterial community composition (Figures 2F and S3C).

### Relationships between the microbial community in the soil‐root continuum and environmental factors

**Figure 3:**
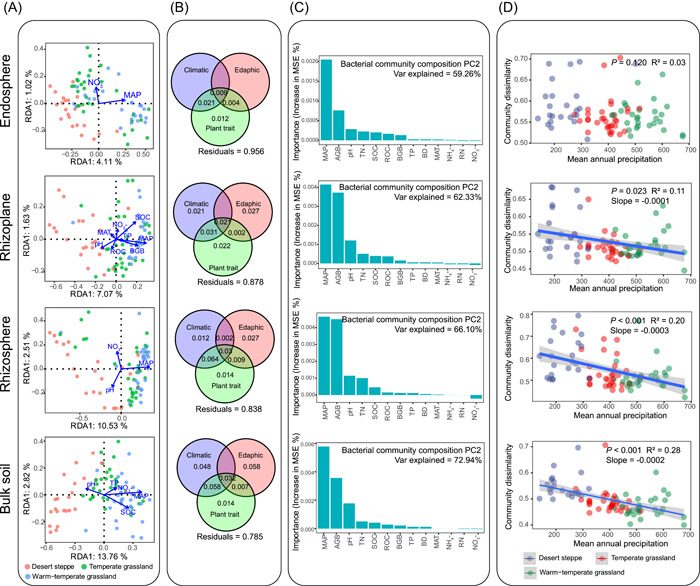
Relationships between the microbial community in the soil‐root continuum and environmental factors. (A) The redundancy analysis (RDA) performed to explore the relationships among the microbial communities, soil properties, and meteorological factors. Only significant factors are shown in the figures; The arrows indicate the lengths and angles between explanatory and response variables and reflect their correlations. (B) Variance partial analysis (VPA) shows the relative explanatory power of soil properties, climate factors, and plant trait groups in determining bacterial community variation across samples in our study. Climatic factors included the altitude, mean annual temperature (MAT), and mean annual precipitation (MAP); edaphic factors included the soil organic carbon (SOC), total nitrogen (TN), nitrate nitrogen (NO_3_^−^), pH, and bulk density (BD); and plant traits included the aboveground biomass (AGB) and belowground biomass (BGB). (C) The importance of environmental factors in predicting the relative abundance of the bacterial community composition (*Y*‐axes [PC2] from the PCoA). Based on the PCoA results, the microhabitats were largely separate in PC1 and the grassland types were mainly separated in PC2, and the importance of environmental factors in predicting the PC1 were shown in Figure S5. (D) The linear regression analysis of the distance of each sample with the mean annual precipitation

Different relationships between the environmental factors and microbial community along the soil‐root continuum were observed (Figure 3A,B), and the contribution of environmental factors in shaping microbial communities gradually declined from the soil (22.93%) and rhizosphere (17.14%) to the endosphere (8.20%; Table S3). All the bacterial communities in the soil‐root continuum showed significant relationships with the MAP (Figure 3A and Table S3). The random forest analysis results showed that the MAP was the most essential environmental factor for all parts of the bacterial community sampled along the soil‐root continuum, followed by the aboveground biomass (AGB) and SOC (Figures 3C and S5). Additionally, we observed significant negative relationships between the MAP and bacterial community distance (unweighted UniFrac distance between each sample site and reference site in the map) in the soil, rhizosphere, and rhizoplane, with a decrease in *R* ^2^ from the soil to the rhizoplane (Figure 3D).

### Microbial recruitment in the soil‐root continuum along the environmental gradient

**Figure 4:**
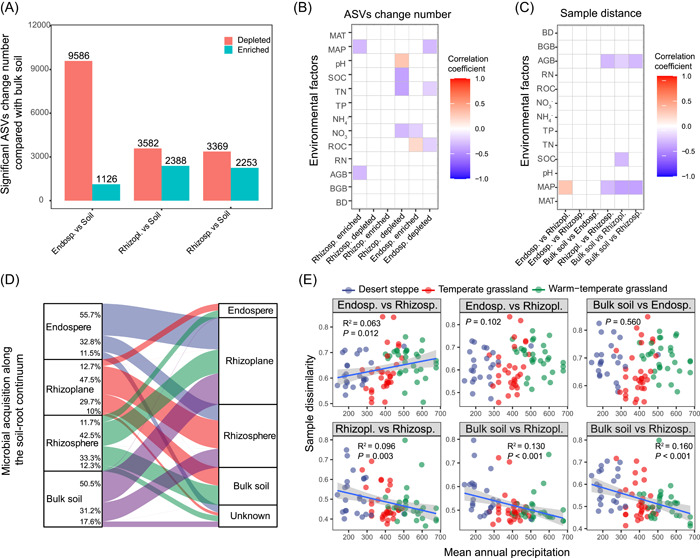
Taxa recruitment in the soil‐root continuum along the environmental gradient. (A) Enriched and depleted bacterial amplicon sequence variant (ASVs) in the endosphere, rhizoplane, and rhizosphere compartments compared with those in the soil. (B) Correlations between enriched and depleted bacterial ASV numbers and environmental factors (*n* = 82). (C) Correlations between the average UniFrac distance of two parts and environmental factors based on the Spearman correlation coefficient. The color represents the value of the Spearman correlation coefficient; red indicates a positive correlation, and blue indicates a negative correlation. Nonsignificant (*p* > 0.05) relationships are shown as blank squares. (D) Taxa similarity based on Source Tracker results is shown to estimate the probability of bacterial taxa derived from the exterior compartments. The rhizosphere, rhizoplane, rhizosphere, and soil were chosen as individual sources. (E) Regression analysis of the dissimilarity of two microhabitats with the mean annual precipitation. Endosp., endosphere; Rhizopl., rhizoplane; Rhizoph., rhizosphere; RN, root nitrogen; ROC, root organic carbon

To identify the amplicon sequence variant (ASV) changes among the microhabitats along the environmental gradient, we conducted differential abundance analyses using a negative binomial distribution with the ASV counts in four microhabitats across site. The soil was selected as the control, and the adjusted *p* value cutoff was set to 0.01. The endosphere had more ASVs depleted than the soil (Figure 4A). Additionally, the differential ASV numbers for each stie showed different relationships with the environmental factors (Figure 4B); for example, the number of enriched ASVs in the rhizosphere was negatively correlated with the MAP, TN, and root C content across sampling sites.

To understand the recruitment dynamics of root‐associated microbial communities with an increase in environmental stress, Source Tracker was used to elucidate potential microbial acquisition along the soil‐root continuum. Most bacterial ASVs in the endosphere were derived from the rhizoplane (55.7%), and only a small part of the rhizoplane bacterial communities (12.7%) was detected in the endosphere compartment (Figure 4C). The rhizosphere community had 33.3% taxa similarity with the soil and 42.5% similarity with the rhizoplane, whereas the rhizoplane community had 29.7% similarity with the soil and 47.5% similarity with the rhizosphere (Figure 4D and Table S4). Additionally, the bacterial community dissimilarities between the rhizosphere and soil, between the rhizoplane and soil, and between the rhizoplane and rhizosphere were negatively correlated with the MAP (Figure 4E). By contrast, the community dissimilarities between the endosphere and rhizosphere were positively correlated with the MAP.

### Assembly mechanisms of microbial communities in the soil‐root continuum along the environmental gradient

**Figure 5:**
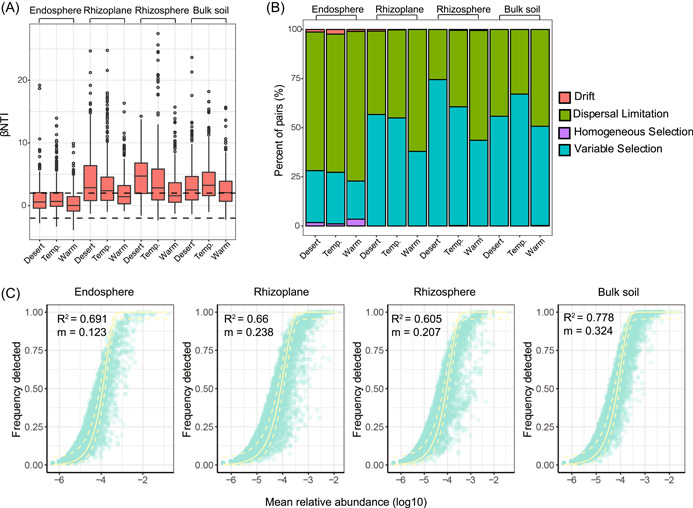
Bacterial community assembly process in the soil‐root continuum along the environmental gradient. (A) βNTI in different microhabitats across the environmental gradient. (B) Null model analysis of the community assembly processes. (C) Fit of Sloan's neutral model for the analysis of community assembly processes. The continuous yellow line represents the best‐fitting neutral model; the dashed lines represent the 95% confidence intervals around the best‐fitting neutral model. *m* indicates the estimated migration rate, and *R* ^2^ indicates the fit to the neutral model

We used the null model and Sloan neutral model to investigate the microbial community assembly process along the soil‐root continuum across the environmental gradient. The null model‐based analysis revealed that stochastic processes (|βNTI| < 2) dominated the community assembly for endosphere bacterial communities. By contrast, deterministic processes (|βNTI| > 2) contributed more to the assembly of the rhizoplane, rhizosphere, and soil communities (Figure 5A,B). Based on the null model results, the variable selection process increased from warm‐temperate to desert grassland for the rhizoplane and rhizosphere communities (Figure 5B). Furthermore, the community assembly also fit the neutral model well (e.g., *R* ^2^ = 0.61–0.77; Figure 5C). The estimated migration rate (*m*) was the highest in the soil and lowest in the endosphere, indicating a decreased dispersal limitation from the endosphere to the soil (Table S5).

### Co‐occurrence networks of microbial taxa in the soil‐root continuum along the environmental gradient

**Figure 6:**
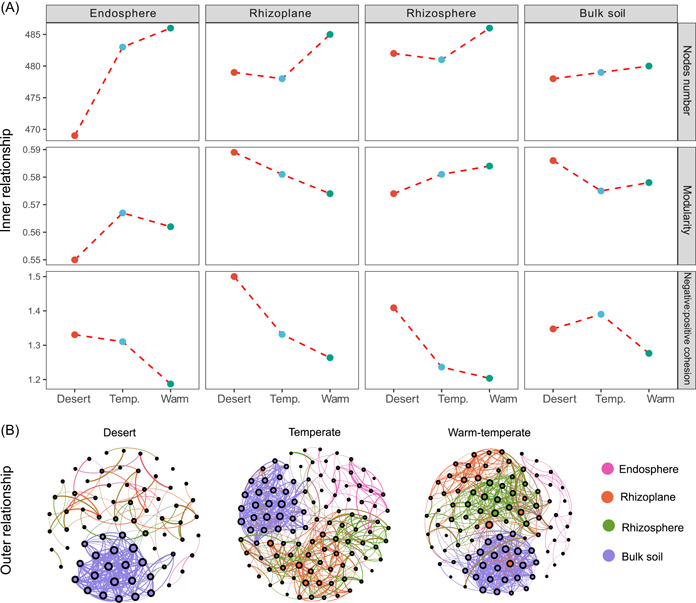
Characterization of microbial network properties within and among microhabitats in different grassland types. (A) The inner relationship panel represents the co‐occurrence networks among taxa within each compartment and grassland type. The node number, modularity, and ratio of negative cohesion to positive cohesion of microbiome networks (SparCC > 0.6, *p* < 0.05) are shown in the figure. The topological properties of the networks are shown in Table S6. Only a single value was calculated for each root compartment in different grassland types. (B) The outer relationship panel represents a network among compartments in different grassland types. Each dot represents one independent sample, and a line connects significant correlated sample pairs (*p* < 0.01) with a Spearman's correlation coefficient >0.6

The properties of co‐occurrence networks for microbial taxa in the soil‐root continuum differed among the grassland types (Figure 6A). The network node number and total edge number among the taxa decreased from warm‐temperate grassland to desert grassland, indicating that the community networks became less complex with an increase in environmental stress (Figure 6A and Table S6). Additionally, we observed different modularities along the soil‐root continuum. For example, the endosphere and rhizosphere networks in the desert grassland showed the lowest modularity, whereas network modularity in the rhizoplane and bulk soil was the highest. However, the ratio of negative cohesion to positive cohesion decreased from the desert grassland to warm‐temperate grassland for the root‐associated microbial community, indicating that more negative associations were observed in the desert grassland. The outer relationships among the microhabitats of ASVs among the soil and root‐associated bacterial communities showed notable differences across different grassland types, with fewer and weaker connections in desert grasslands (Figure 6B and Table S6).

## DISCUSSION

In the present study, we observed different patterns of bacterial community diversity, composition, assembly, and co‐occurrence networks in the soil‐root continuum along an environmental gradient that spanned from warm‐temperate grassland to desert grassland. Overall, we found that (1) microhabitats defined by their proximities to plant roots were more important than the grassland type in shaping microbial community assembly, and the influence of environmental factors on the microbial community decreased from the soil to the endosphere; (2) environmental stress increased the dissimilarity of microbial communities from the rhizosphere and rhizoplane to bulk soil; (3) deterministic processes with variable selection were more important in the rhizosphere and rhizoplane, increasing from warm‐temperate grassland to desert grassland; and (4) co‐occurrence networks of the root‐associated microbial community had a higher negative cohesion to positive cohesion ratio in desert grasslands.

### Effects of microhabitats and environmental factors on the microbial community in the soil‐root continuum along an environmental gradient

As expected, the diversity and composition of microbial communities at the fine‐scale were substantially affected by microhabitats along the soil‐root continuum (Figure 2 and Table S2). The lower diversity in the endosphere than in the rhizosphere and soil is likely due to selective filtering from the outside of the root to the inside of the root; these patterns are consistent with observations in rice [2], *Arabidopsis* [17], and mangroves [27]. Additionally, the lowest bacterial diversity in the desert grassland suggests that selective filtering is also mediated by environmental factors and may be stronger with decreases in precipitation, soil nutrient availability, and SOC (Table S1 and Figure S6), consistent with the study findings on drylands by Maestre et al. [1] and Jiao et al. [39].

The microhabitats explained the most variation in bacterial community structure (Figure 2D), highlighting the importance of microhabitats in driving bacterial community composition and function in the soil‐root continuum [27, 40, 41]. However, these results differed from those of a rice bacterial microbiome study, in which the study site was the largest source of microbial community variation (30%) rather than microhabitats (21%) [2]. These differences might be attributed to the limited number and geological distance among the field sites in that study (maximum separation of ∼125 km and eight fields examined in the rice study) and the intrinsic differences between natural grasslands and cultivated rice paddies. Additionally, previous studies have reported that both plant variety types and species demonstrate markedly affect the microbial community composition in the soil‐root continuum under greenhouse conditions [17, 41, 42, 43], but we found that host plants only impacted the endosphere communities across spatially broad environmental gradients (Figure S4).

On the Loess Plateau, the MAP and soil nutrient levels decreased from warm‐temperate grasslands to desert grasslands, suggesting higher environmental stress in desert grasslands (Table S1). Soil microbial community composition and function are often related to SOC [44] and are limited by C in dryland soils [45, 46], but we determined that MAP was the most important environmental constraint on community assembly along the soil‐root continuum in this region. Furthermore, we observed that the total constraint of environmental factors on the microbial community decreased from the soil to the endosphere (Figures 3 and S5, and Table S3). The above results indicate that the soil and rhizosphere communities are more sensitive to the fluctuations in the soil environment, whereas the endosphere communities are less responsive to environmental variation, likely because of a more buffered and stable environment in the root epidermis [1, 13].

### Taxon recruitment and community assembly processes in the soil‐root continuum are influenced by environmental factors

Generally, plant roots assemble their microbiomes in two steps: the first step involves recruitment to the vicinity of the root and the second step involves entry into the root of microbial species‐specific genetic factors [6]. In our study, decreasing proportions of taxa in the endosphere originating from the rhizoplane or rhizosphere were observed, suggesting that a subset of microbes initially recruited to the rhizosphere are bound to the rhizoplane and are selectively filtered by direct physical association with the root, significantly affecting community assembly in the endosphere [2]. Additionally, our results support that environmental factors affect the recruitment of taxa from the soil to the endosphere (Figure 4A,B) [47, 48]. We also demonstrated that the MAP was positively correlated with community dissimilarities between the endosphere and rhizosphere and was negatively correlated with community dissimilarities between the soil and rhizosphere and rhizoplane microbial communities (Figure 4D,E and Table S4). The lower number of taxa in the endosphere that originated from outside the root and higher dissimilarity among root‐associated communities in the desert grasslands might be associated with low connectivity and less dispersion in drier soils, as well as a potentially stronger microenvironmental gradient from the root to the bulk soil defined by water, pH, and nutrients in soils with less MAP [49]. These results infer that tighter coupling of the rhizoplane and endosphere communities at sites with lower precipitation reflects coevolution between plants and root endophytes that could facilitate nutrient uptake or minimize host stress.

Environmental shifts could cause deterministic and stochastic assembly process changes and drive the spatial distribution of microbial communities [25]. The soil microbial assembly processes across environmental gradients have been investigated in previous studies [28, 50], but assembly processes in the soil‐root continuum along environmental gradients are lacking. In the present study, stochastic assembly processes were dominant in endosphere communities (Figure 5A,B), in contrast to the finding of Zhuang et al. [27], who observed the dominance of deterministic processes in the endosphere microbiomes of mangroves. Meanwhile, the soil, rhizosphere, and rhizoplane communities were governed primarily by variable selection of deterministic processes that increased in the rhizosphere and rhizoplane with increases in environmental stress, consistent with the results of the neutral model of a decreased estimated migration rate (*m* value) from bulk soil to the endosphere, as higher *m* values indicate that microbial communities are less dispersal limited [51, 52] (Figure 5B,D). A community under strong environmental selection often has low dispersal limitation because selection will result in microbial communities comprising a small number of highly abundant species, with large variation in the birth and death rates of rare taxa within the communities [53]. Our study highlights the strong effects of environmental factors on rhizosphere and rhizoplane microbial assembly processes.

### Different co‐occurrence networks for microbial taxa in the soil‐root continuum among the grassland types

An increasing body of evidence has predicted that ecological networks comprising higher ratios of negative to positive associations between taxa are more stable to environmental change [34, 54] because negative interactions minimize co‐oscillation in communities under disturbance [34, 55, 56]. Besides, higher modularity in a given network could indicate stabilized communities by restricting the impact of losing a taxa to its own module, preventing the effects of that taxon's extinction from propagating to affect the remainder of the network [57]. A previous study showed that environmental stress destabilizes microbial community networks, and communities dominated by positive co‐occurrences occur more frequently in high‐stress environments [58]. de Vries et al. [33] reported that the long‐lasting legacy effect of drought promoted destabilizing properties in soil bacterial community networks in the soil dominated by a fast‐growth grass in a manipulated experiment. However, we found that the ratios of negative to positive associations decreased from the desert grassland to warm‐temperate grassland in endosphere, rhizoplane, and rhizosphere communities, and modularity also decreased from the desert to warm‐temperate grasslands in the rhizoplane and bulk soil network (Figure 6A and Table S6), indicating that these destabilizing properties in network microbial communities in the soil‐root continuum for desert grasslands were not observed in our study (Figure 6).

Differences between our results and past observations might be explained by the vegetation types examined [58] or that we sampled microbiomes in situ under a natural environment gradient instead of using a manipulated field experiment (e.g., [33]). The conditions in the natural desert grassland likely function as a deterministic filtering factor to select certain microbes adapted to prolonged low precipitation and soil nutrient environment. Consequently, microbial network structures should reflect stress conditions with weaker and more negative connections. This finding is consistent with that of Yuan et al. [59], who reported that thermal acclimation of soil microbes to long‐term warming enhances network stability. The weaker relationships within the root‐associated community suggest that the microbial community may be less responsive to environmental change in the desert grassland than in the temperate or warm‐temperate grassland, a finding that may benefit the microbes and plants to resist environmental disturbance in the desert grassland where soil moisture and nutrients often limit plant and microbial growth.

## CONCLUSIONS

In the present study, we investigated the microbial community diversity, community assembly characteristics, and their co‐occurrence networks in the soil‐root continuum at 82 field sites along an environmental gradient (MAP and soil nutrients) in the semiarid and arid Loess Plateau. We found that the microhabitat was the largest source of the variations in bacterial diversity and community composition, and environmental factors impacted the microbial community in the soil, rhizosphere and rhizoplane, but their effects decreased along the soil‐root continuum. Additionally, increasing environmental stress likely causes higher selective filtering from outside the root to inside the root, as evidenced by a decoupling of relationships between the soil and rhizosphere and rhizoplane microbiomes but strengthened linkages between the endosphere and rhizosphere communities at locations with less precipitation. Furthermore, we found that deterministic processes with variable selection were increased from the warm‐temperate grassland to desert grassland, particularly in the rhizosphere and rhizoplane communities, and co‐occurrence networks of the soil and root‐associated microbes were not destabilized in desert grassland with a history of prolonged low precipitation and soil nutrients. The present study provides new insights into understanding the microbial diversity and assembly mechanisms of the bacterial community in the soil‐root continuum along an environmental gradient in the Loess Plateau and fills a critical gap in our understanding of soil–plant–microbe interactions across broad regional scales.

## MATERIALS AND METHODS

### Study area

The Loess Plateau covers an area of 640,000 km^2^ in the upper and middle reaches of the Yellow River basin in Northern China (Figure 1), which experiences arid and semiarid climate conditions with a mean annual temperature (MAT) ranging from 4.3°C to 14.3°C and a MAP ranging from 160 to 750 mm from the northwest to southeast. This study mainly focuses on the grasslands of the Loess Plateau, which cover 42.86% of the total area [60].

### Field survey and soil sample collection

Based on grassland inventory data collected across the Loess Plateau, 233 grassland sites were surveyed in the summer from 2011 to 2013. Among these sites, we chose 82 of the most representative natural grasslands (22 desert steppes, 32 temperate grasslands, and 28 warm‐temperate grasslands) based on climate and vegetation characteristics (Figure 1A, B) [61]. The sites spanned a longitudinal range from 103°E to 112°E and a latitudinal range from 34°N to 40°N; we resurveyed these sites in July–August 2018. The MAT and MAP of the last 30 years are collected from 64 meteorological stations on the Loess Plateau and are available from the National Climate Centre of the China Meteorological Administration (www.nmic.gov.cn). At each sampling site, we sketched a 100 m transect to identify a representative section and established four 1 × 1 m quadrats at 20 m intervals. The aboveground parts of green plants (AGB) and belowground biomass (BGB) were then collected and dried at 65°C for biomass determination by weight. At each site, a composite soil sample from four plots (five samples per plot) (0 ~20 cm depth) was collected. The samples were passed through a 2 mm sieve to remove the roots and other debris. After field collection, each soil sample was separated into two subsamples. One subsample was immediately frozen and stored in liquid nitrogen for molecular analyses, while the other subsample was air‐dried for chemical analyses.

### Sample collection of rhizosphere, rhizoplane, and endosphere fractions

At each site, the roots of the most dominant grass species were collected. In the desert steppe, the major species are *Stipa* spp. and *Reaumuria Linn*.; temperate grasslands are dominated by *Stipa* spp. and *Agropyron cristatum*; warm temperate grasslands are dominated by *Artemisia sacrorum*. Excess soil was manually shaken from the roots, leaving approximately 1 mm of soil still attached to the roots (Figure 1C). The roots were cut into approximately 5 cm segments using sterile gloves and sterile scissors, stored in a 50 ml tube, and transferred to liquid nitrogen. These roots were transported to the laboratory to isolate the rhizosphere, rhizoplane, and endosphere as described previously in Edwards et al. [2] and Durán et al. [17]. Briefly, when the samples were transferred to the laboratory, we directly separated the soil of the rhizosphere from the roots by placing them in a sterile flask with 50 ml of sterile phosphate‐buffered saline (PBS) solution. The roots were then stirred vigorously with sterile forceps to clean all the soil from the root surfaces. The soil suspension stripped from the roots was poured into a 50 ml Falcon tube and stored as the rhizosphere compartment at −80°C until DNA extraction. The roots designated for rhizoplane collection were cleaned in the laboratory and placed in a Falcon tube with 15 ml of PBS. Tightly adhered microbes at the root surface were removed using a sonication protocol that was originally developed for plant roots. The roots in the Falcon tube were sonicated for 30 s in the range of 50–60 Hz. The roots were then removed, and the liquid PBS fraction was kept as the rhizoplane compartment. The roots designated for endosphere collection were cleaned and sonicated as previously described. Two additional sonication procedures using clean PBS solution were performed to ensure that all the microbes were removed from the root surface. The sonicated roots were then stored at −80°C until DNA extraction on the same day. In total, four parts of the soil and root‐associated microbial communities were collected at 82 sites, resulting in 328 DNA samples.

### Measurements of the physicochemical properties of soil and plants

The physicochemical properties of the soil, including soil pH, soil organic carbon (SOC), total nitrogen (TN), NH_4_^+^ and NO_3_^−^ and total phosphorus (TP), were quantified as previously reported [62]. The plant aboveground C content was measured using dichromate oxidation [63], and the N content was measured using the Kjeldahl method [64]. All the soil and plant physicochemical measurements were performed in duplicate. The climate factors, edaphic factors, and plant traits in each grassland type are shown in Table S1.

### DNA extraction, ion S5 XL sequencing, and data processing

Microbial DNA was extracted from 0.5 g of soil samples (or 1 g of root samples) using an E.Z.N.A. Soil DNA kit (Omega Biotek) according to the manufacturer's protocol. The V4 region of the bacterial and archaeal 16S rRNA gene was polymerase chain reaction (PCR) amplified (95°C for 2 min followed by 27 cycles of 95°C for 30 s, 55°C for 30 s, and 72°C for 45 s, with a final extension at 72°C for 10 min) using the primers 515 F (5′‐barcode‐GTGYCAGCMGCCGCGGTAA‐3′) and 806 R (5′‐GGACTACNVGGGTWTCTAAT‐3′) [42]. All PCRs were performed using the Phusion® High‐Fidelity PCR Master Mix (New England Biolabs). Sequencing libraries were generated using the Ion Plus Fragment Library Kit 48 rxns (Thermo Scientific) following the manufacturer's recommendations. The library quality was assessed using a Qubit@ 2.0 Fluorometer (Thermo Scientific) and then sequenced on an Ion S5™ XL platform.

Reads from 16S‐V4 sequencing were analyzed using QIIME 2 (v2018.4) [65]. Reads from 16S‐V4 were trimmed where the average quality score dropped below 25 and were dereplicated using DADA2 as implemented in QIIME 2 [66] with a paired‐end setting (including quality control, trimming, pair‐joining, and chimera removals), resulting in 91.96% retained reads. The 16S‐V4 representative ASVs were assigned taxonomy using the SILVA 128 database [66] and naïve Bayes classifier in QIIME 2 [67] to produce taxonomy tables. Representative sequences, taxonomy, and count tables from bacterial and fungal reads were merged in QIIME 2. Phylogenetic trees were built in QIIME 2 using the MAFFT alignment in QIIME 2 and FastTree algorithm [68]. More than 70,000 high‐quality 16S rRNA gene sequences were obtained per sample, and 273 archaeal ASVs and 60211 bacterial ASVs were detected. The archaeal and bacterial 16S rRNA gene sequencing data were uploaded to the NCBI SRA database under accession number PRJNA561671.

### Calculations and statistical analyses

All statistical analyses were performed using the R software package (version 3.5) [69]. Part of data were visualized online by using ImageGP [70] and EVenn [71]. Similarities among samples in terms of ASVs were determined using the unweighted and weighted UniFrac distance calculated using the ASV phylogenetic relationships with QIIME 2. The microbial taxa composition was compared using principal coordinate analysis (PCoA) based on the dissimilarity distance. Permutational‐based analysis of variance was used to test the significance of each subset group (adonis function in the “vegan” package [72]). We used a canonical analysis of principal coordinates (CAP) to better quantify the influence of microhabitats and grassland types on beta diversity using the “CAPdiscrim” function in the R “BiodiversityR” package [73]. Distance‐based redundancy analysis (dbRDA) was performed to elucidate the relationships between microbial communities and environmental factors, and vif.cca() was applied to test for collinearity among environmental factors [73]. Variance partial analysis (VPA) in the vegan package was performed to quantify the amount of variation explained by each primary environmental factor. To determine which environmental factors were essential, we regressed the bacterial and archaeal ASVs against environmental factors using default parameters of an R‐implemented algorithm (R package “randomForest,” ntree = 1000, using a default mtry of *p*/3, where *p* is the number of taxa in the class) [74]. Additionally, we performed linear regression to test for relationships between the MAP and distances (unweighted UniFrac distance between each sample site with a reference site [with “*” mask in map]) of the soil, rhizosphere, rhizoplane, and endosphere communities.

Variation in phylogenetic diversity was quantified as null model‐based phylogenetic β‐diversity to test for the various community assembly processes [75, 76]. We applied βNTI combined with Bray–Curtis‐based Raup–Crick (RC_bray_) [76] to quantify the contribution of major ecological processes to the assembly of root‐associated microbial communities. The percentage value was derived from the statistical average of the ecological process results. The Sloan neutral community model [52] was selected to determine the contribution of stochastic processes to microbial community assembly by predicting the relationship between the frequency with which taxa occur in a set of local communities (proportion of local communities in which each taxon is detected) and their abundance in the metacommunity (estimated by the mean relative abundance across all local communities within biomes or clusters). We used the fit of the neutral model (*R* ^2^) to infer the stochastic processes. The *m* value conveys the estimated migration rate; higher *m* values indicate that microbial communities are less dispersal limited [51, 52].

The inner community similarity (common taxa) of each microhabitat was also characterized using Source Tracker, a Bayesian approach to estimate the proportion of contaminants in a given community that originate from other source environments [77]. We separately analyzed bacterial networks for different microhabitats in different grassland types as well as their network associations among them. The inner relationship (networks among taxa within each microhabitat and grassland type) was based on a network co‐occurrence analysis of ASVs with relative abundance >0.001. Each dot represents a bacterial or archaeal phylotype (an ASV clustered at 97%), and the links represent statistically significant (*p* < 0.01) SparCC correlations with a correlation coefficient >0.6 in each respective microhabitat. The outer relationship was based on a network co‐occurrence analysis of relationships linking communities among compartments for each grassland type. Each dot represents one independent sample, and the connection represents a statistically significant (*p* < 0.01) Spearman's correlation with a correlation coefficient >0.6. Additionally, the number of nodes and edges, average path length, network diameter, cumulative degree distribution, clustering coefficient, and modularity were calculated according to a previous study [78] using the function “network()” in the R package “ggClusterNet” [79]. The networks were visualized using the interactive platform Gephi [80]. The map was generated using ArcMap Version 10.0 (http://www.esri.com/).

## AUTHOR CONTRIBUTIONS

Yangquanwei Zhong and Weiming Yan conceptualized the research program, designed the experiments, and coordinated the project. Guangyu Zhu, Jin Liu, Xiaoyu Jia, and Weiming Yan collected the root and soil samples in the field and performed the soil physical and chemical property analyses. Yangquanwei Zhong and Patrick O. Sorensen performed the data analyses. Yangquanwei Zhong, Weiming Yan, Patrick O. Sorensen, Ruiwu Wang, and Zhouping Shangguan drafted the manuscript. All the authors were involved in critically revising and approving the final version.

## CONFLICTS OF INTEREST

The authors declare no conflicts of interest.

## Supporting information

Supplementary information.

Supplementary information.

## Data Availability

All the data needed to evaluate the conclusions in the paper are presented in the paper and/or the supplementary materials. The 16S and sequences were submitted to the SRA of the NCBI under accession number PRJNA561671. The scripts used in this study can be found at https://github.com/zhongyqw/Root‐microbiota‐along‐an‐environmental‐gradient. Supporting information (graphical abstract, slides, videos, Chinese translated version, and updated materials) is available online DOI or http://www.imeta.science.

## References

[1] Maestre, Fernando T. , Manuel Delgado‐Baquerizo , Thomas C. Jeffries , David J. Eldridge , Victoria Ochoa , Beatriz Gozalo , José Luis Quero , et al. 2015. “Increasing Aridity Reduces Soil Microbial Diversity and Abundance in Global Drylands. Proceedings of the National Academy of Sciences of the United States of America 112: 15684–9. 10.1073/pnas.1516684112 26647180 PMC4697385

[2] Edwards, Joseph , Cameron Johnson , Christian Santos‐Medellin , Eugene Lurie , Natraj K. Podishetty , Srijak Bhatnagar , Jonathan A. Eisen , et al. 2015. “Structure, Variation, and Assembly of the Root‐Associated Microbiomes of Rice.” Proceedings of the National Academy of Sciences of the United States of America 112: E911–20. 10.1073/pnas.1414592112 25605935 PMC4345613

[3] Haichar, Fethel Z. , Christine Marol , Odile Berge , J. Ignacio Rangel‐Castro , James I. Prosser , Jérôme Balesdent , Thierry Heulin , et al. 2008. “Plant Host Habitat and Root Exudates Shape Soil Bacterial Community Structure.” The ISME Journal 2: 1221–30. 10.1038/ismej.2008.80 18754043

[4] Reinhold‐Hurek, Barbara , Wiebke Bunger , Claudia S. Burbano , Mugdha Sabale , and Thomas Hurek . 2015. “Roots Shaping Their Microbiome: Global Hotspots for Microbial Activity.” Annual Review of Phytopathology 53: 403–24. 10.1146/annurev-phyto-082712-102342 26243728

[5] Lundberg, Derek S. , Sarah L. Lebeis , Sur H. Paredes , Scott Yourstone , Jase Gehring , Stephanie Malfatti , Julien Tremblay , et al. 2012. “Defining the Core Arabidopsis Thaliana Root Microbiome.” Nature 488: 86–90. 10.1038/nature11237 22859206 PMC4074413

[6] Bulgarelli, Davide , Klaus Schlaeppi , Stijn Spaepen , Emiel V.L. van Themaat , and Paul Schulze‐Lefert . 2013. “Structure and Functions of the Bacterial Microbiota of Plants.” Annual Review of Plant Biology 64: 807–38. 10.1146/annurev-arplant-050312-120106 23373698

[7] Philippot, Laurent , Jos M. Raaijmakers , Philippe Lemanceau , and Wim H. van der Putten . 2013. “Going Back to the Roots: The Microbial Ecology of the Rhizosphere.” Nature Reviews Microbiology 11: 789–99. 10.1038/nrmicro3109 24056930

[8] Schlaeppi, Klaus , Nina Dombrowski , Ruben G. Oter , Emiel V. L. van Themaat , and Paul Schulze‐Lefert . 2014. “Quantitative Divergence of the Bacterial Root Microbiota in *Arabidopsis thaliana* Relatives.” Proceedings of the National Academy of Sciences of the United States of America 111: 585–92. 10.1073/pnas.1321597111 24379374 PMC3896156

[9] Sorensen, Patrick O. , Jennifer M. Bhatnagar , Lynn Christenson , Jorge Duran , Timothy Fahey , Melany C. Fisk , Adrien C. Finzi , et al. 2019. “Roots Mediate the Effects of Snowpack Decline on Soil Bacteria, Fungi, and Nitrogen Cycling in a Northern Hardwood Forest.” Frontiers in Microbiology 10: 926. 10.3389/fmicb.2019.00926 31114563 PMC6503048

[10] Classen, Aimée T. , Maja K. Sundqvist , Jeremiah A. Henning , Gregory S. Newman , Jessica A. M. Moore , Melissa A. Cregger , Leigh C. Moorhead , et al. 2015. “Direct and Indirect Effects of Climate Change on Soil Microbial and Soil Microbial‐Plant Interactions: What Lies Ahead?” Ecosphere 6: 1–21. 10.1890/ES15-00217.1

[11] Aslani, Farzad , Leho Tedersoo , Sergei Põlme , Oliver Knox , and Mohammad Bahram . 2020. “Global Patterns and Determinants of Bacterial Communities Associated with Ectomycorrhizal Root Tips of Alnus Species.” Soil Biology and Biochemistry 148: 107923. 10.1016/j.soilbio.2020.107923

[12] Lauber, Christian L. , Micah Hamady , Rob Knight , and Noah Fierer . 2009. “Pyrosequencing‐based Assessment of Soil pH as a Predictor of Soil Bacterial Community Structure at the Continental Scale.” Applied and Environmental Microbiology 75: 5111–20. 10.1128/Aem.00335-09 19502440 PMC2725504

[13] Bahram, Mohammad , Falk Hildebrand , Sofia K. Forslund , Jennifer L. Anderson , Nadejda A. Soudzilovskaia , Peter M. Bodegom , Johan Bengtsson‐Palme , et al. 2018. “Structure and Function of the Global Topsoil Microbiome.” Nature 560: 233–7. 10.1038/s41586-018-0386-6 30069051

[14] Delgado‐Baquerizo, Manuel , Angela M. Oliverio , Tess E. Breweralberto , Benavent‐González Alberto , David J. Eldridge , Richard D. Bardgett , and Fernando T. Maestre , et al. 2018“A Global Atlas of the Dominant Bacteria Found in Soil.” Science 359: 320–5. 10.1126/science.aap9516 29348236

[15] Makhalanyane, Thulani P. , Angel Valverde , David Velázquez , Eoin Gunnigle , Marc W. Van Goethem , Antonio Quesada , and Don A. Cowan . 2015. “Ecology and Biogeochemistry of Cyanobacteria in Soils, Permafrost, Aquatic and Cryptic Polar Habitats.” Biodiversity and Conservation 24: 819–40. 10.1007/s10531-015-0902-z

[16] Nuccio, Erin E. , James Anderson‐Furgeson , Katerina Y. Estera , Jennifer Pett‐Ridge , Perry de Valpine , Eoin L. Brodie , and Mary K. Firestone . 2016. “Climate and Edaphic Controllers Influence Rhizosphere Community Assembly for a Wild Annual Grass.” Ecology 97: 1307–18. 10.1890/15-0882.1 27349106

[17] Durán, Paloma , Thorsten Thiergart , Ruben Garrido‐Oter , Matthew Agler , Eric Kemen , Paul Schulze‐Lefert , and Stéphane Hacquard . 2018. “Microbial Interkingdom Interactions in Roots Promote Arabidopsis Survival.” Cell 175: 973–83. 10.1016/j.cell.2018.10.020 30388454 PMC6218654

[18] Walters, William A. , Zhao Jin , Nicholas Youngblut , Jason G. Wallace , Jessica Sutter , Wei Zhang , Antonio González‐Peña , et al. 2018. “Large‐Scale Replicated Field Study of Maize Rhizosphere Identifies Heritable Microbes.” Proceedings of the National Academy of Sciences of the United States of America 115: 7368–73. 10.1073/pnas.1800918115 29941552 PMC6048482

[19] Zhalnina, Kateryna , Katherine B. Louie , Zhao Hao , Nasim Mansoori , Ulisses N. da Rocha , Shengjing Shi , and Cho Heejung , et al. 2018. “Dynamic Root Exudate Chemistry and Microbial Substrate Preferences Drive Patterns in Rhizosphere Microbial Community Assembly.” Nature Microbiology 3: 470–80. 10.1038/s41564-018-0129-3 29556109

[20] Karray, Fatma , Mahmoud Gargouri , Asma Chebaane , Najla Mhiri , Ahmed Mliki , and Sami Sayadi . 2020. “Climatic Aridity Gradient Modulates the Diversity of the Rhizosphere and Endosphere Bacterial Microbiomes of *Opuntia Ficus‐Indica* .” Frontiers in Microbiology 11: 1622. 10.3389/fmicb.2020.01622 32849335 PMC7401614

[21] Praeg, Nadine , Harald Pauli , and Paul Illmer . 2019. “Microbial Diversity in Bulk and Rhizosphere Soil of *Ranunculus glacialis* Along a High‐Alpine Altitudinal Gradient.” Frontiers in Microbiology 10: 1429. 10.3389/fmicb.2019.01429 31338073 PMC6629913

[22] Wang, Xiaobo , Jing Y. Xiaotao Lü , Ye D. Zhengwen Wang , Jizhong Z. Weixin Cheng , et al. 2017. “Habitat‐specific Patterns and Drivers of Bacterial β‐Diversity in China's Drylands.” The ISME Journal 11: 1345–58. 10.1038/ismej.2017.11 28282041 PMC5437346

[23] Martiny, Jennifer B. H. , Jonathan A. Eisen , Kevin Penn , Steven D. Allison , and M. Claire Horner‐Devine . 2011. “Drivers of Bacterial β‐Diversity Depend on Spatial Scale.” Proceedings of the National Academy of Sciences of the United States of America 108: 7850–4. 10.1073/pnas.1016308108 21518859 PMC3093525

[24] Vellend, Mark , and Anurag Agrawal . 2010. “Conceptual Synthesis in Community Ecology.” The Quarterly Review of Biology 85: 183–206. 10.1086/652373 20565040

[25] Chase, Jonathan M. , and Jonathan A. Myers . 2011. “Disentangling the Importance of Ecological Niches From Stochastic Processes Across Scales.” Philosophical Transactions of the Royal Society B: Biological Sciences 366: 2351–63. 10.1098/rstb.2011.0063 PMC3130433 21768151

[26] Hubbell, Stephen P. 2011. The Unified Neutral Theory of Biodiversity and Biogeography (MPB‐32). Princeton, NJ: Princeton University Press. 10.1515/9781400837526 21561679

[27] Zhuang, Wei , Xiaoli Yu , Ruiwen Hu , Zhiwen Luo , Xingyu Liu , Xiafei Zheng , and Fanshu Xiao , et al. 2020. “Diversity, Function and Assembly of Mangrove Root‐Associated Microbial Communities at a Continuous Fine‐scale.” NPJ Biofilms and Microbiomes 6: 1–10. 10.1038/s41522-020-00164-6 33184266 PMC7665043

[28] Jiao, Shuo , Weimin Chen , and Gehong Wei . 2021. “Linking Phylogenetic Niche Conservatism to Soil Archaeal Biogeography, Community Assembly and Species Coexistence.” Global Ecology and Biogeography 30: 1488–501. 10.1111/geb.13313

[29] Tripathi, Binu M. , Mincheol Kim , Ang Lai‐Hoe , Nor A. A. Shukor , Raha A. Rahim , Rusea Go , and Jonathan M. Adams . 2013. “pH Dominates Variation in Tropical Soil Archaeal Diversity and Community Structure.” FEMS Microbiology Ecology 86: 303–11. 10.1111/1574-6941.12163 23773164

[30] Mamet, Steven D. , Ellen Redlick , Michelle Brabant , Eric G. Lamb , Bobbi L. Helgason , Kevin Stanley , and Steven D. Siciliano . 2019. “Structural Equation Modeling of a Winnowed Soil Microbiome Identifies How Invasive Plants Re‐Structure Microbial Networks.” The ISME Journal 13: 1988–96. 10.1038/s41396-019-0407-y 30926920 PMC6776034

[31] Wei, Zhong , Tianjie Yang , Ville‐Petri Friman , Yangchun Xu , Qirong Shen , and Alexandre Jousset . 2015. “Trophic Network Architecture of Root‐Associated Bacterial Communities Determines Pathogen Invasion and Plant Health.” Nature Communications 6: 1–9. 10.1038/ncomms9413 PMC4598729 26400552

[32] Ramirez, Kelly S. , Stefan Geisen , Elly Morriën , Basten L. Snoek , and Wim H. van der Putten . 2018. “Network Analyses Can Advance Above‐Belowground Ecology.” Trends in Plant Science 23: 759–68. 10.1016/j.tplants.2018.06.009 30072227

[33] de Vries, Franciska T. , Rob I. Griffiths , Mark Bailey , Hayley Craig , Mariangela Girlanda , Hyun S. Gweon , Sara Hallin , et al. 2018. “Soil Bacterial Networks are Less Stable Under Drought Than Fungal Networks.” Nature Communications 9: 3033. 10.1038/s41467-018-05516-7 PMC6072794 30072764

[34] Coyte, Katharine Z. , Jonas Schluter , and Kevin R. Foster . 2015. “The Ecology of the Microbiome: Networks, Competition, and Stability.” Science 350: 663–6. 10.1126/science.aad2602 26542567

[35] Shi, Shengjing , Erin E. Nuccio , Zhou J. Shi , Zhili He , Jizhong Zhou , and Mary K. Firestone . 2016. “The Interconnected Rhizosphere: High Network Complexity Dominates Rhizosphere Assemblages.” Ecology Letters 19: 926–36. 10.1111/ele.12630 27264635

[36] Feng, Xiaoming , Bojie Fu , Shilong Piao , Shuai Wang , Philippe Ciais , Zhenzhong Zeng , Yihe Lü , et al. 2016. “Revegetation in China's Loess Plateau is Approaching Sustainable Water Resource Limits.” Nature Climate Change 6: 1019–22. 10.1038/nclimate3092

[37] Xin, Zhongbao , Jiongxin Xu , and Wei Zheng . 2008. “Spatiotemporal Variations of Vegetation Cover on the Chinese Loess Plateau (1981‐2006): Impacts of Climate Changes and Human Activities.” Science in China Series D‐Earth Sciences 51: 67–78. 10.1007/s11430-007-0137-2

[38] Zhong, Yangquanwei , Jin Liu , Xiaoyu Jia , Zhuangsheng Tang , Zhouping Shangguan , Ruiwu Wang , and Yan Weiming . 2022. “Environmental Stress‐Discriminatory Taxa are Associated with High C and N Cycling Functional Potentials in Dryland Grasslands.” Science of The Total Environment 817: 152991. 10.1016/j.scitotenv.2022.152991 35026259

[39] Jiao, Shuo , Haiyan Chu , Baogang Zhang , Xiaorong Wei , Weimin Chen , and Gehong Wei . 2022. “Linking Soil Fungi to Bacterial Community Assembly in Arid Ecosystems.” iMeta 1: e2. 10.1002/imt2.2 PMC10989902 38867731

[40] Lundberg, Derek S. , Sarah L. Lebeis , Sur H. Paredes , Scott Yourstone , Jase Gehring , Stephanie Malfatti , Julien Tremblay , et al. 2012. “Defining the Core *Arabidopsis thaliana* Root Microbiome.” Nature 488: 86–90. 10.1038/nature1123 22859206 PMC4074413

[41] Jin, Tao , Yayu Wang , Yueying Huang , Jin Xu , Pengfan Zhang , Nian Wang , Liu Xin , et al. 2017. “Taxonomic Structure and Functional Association of Foxtail Millet Root Microbiome.” Gigascience 6: 1–12. 10.1093/gigascience/gix089 PMC7059795 29050374

[42] Walters, William , Embriette R. Hyde , Donna Berg‐Lyons , Gail Ackermann , Greg Humphrey , Alma Parada , Jack A. Gilbert , et al. 2016. “Improved Bacterial 16S rRNA Gene (V4 and V4‐5) and Fungal Internal Transcribed Spacer Marker Gene Primers for Microbial Community Surveys.” Msystems 1: e00009–15. 10.1128/mSystems.00009-15 PMC5069754 27822518

[43] Zhang, Jingying , Yong‐Xin Liu , Na Zhang , Bin Hu , Tao Jin , Haoran Xu , Qin Yuan , et al. 2019. “NRT1.1B is Associated with Root Microbiota Composition and Nitrogen Use in Field‐Grown Rice.” Nature Biotechnology 37: 676–84. 10.1038/s41587-019-0104-4 31036930

[44] Siciliano, Steven D. , Anne S. Palmer , Tristrom Winsley , Eric Lamb , Andrew Bissett , Mark V. Brown , Josie van Dorst , et al. 2014. “Soil Fertility is Associated With Fungal and Bacterial Richness, Whereas pH is Associated with Community Composition in Polar Soil Microbial Communities.” Soil Biology and Biochemistry 78: 10–20. 10.1016/j.soilbio.2014.07.005

[45] Manzoni, Stefano , Joshua P. Schimel , and Amilcare Porporato . 2012. “Responses of Soil Microbial Communities to Water Stress: Results From a Meta‐Analysis.” Ecology 93: 930–8. 10.1890/11-0026.1 22690643

[46] Angel, Roey , Zohar Pasternak , M., Ines , M., Soares , Ralf, Conrad1 , and Osnat Gillor . 2013. “Active and Total Prokaryotic Communities in Dryland Soils.” FEMS Microbiology Ecology 86: 130–8. 10.1111/1574-6941.12155 23730745

[47] Wu, Ai‐Lian , Xiao‐Yan Jiao , Jin‐Song Wang , Er‐Wei Dong , Jun Guo , Li‐Ge Wang , Sun An‐Qi , et al. 2021. “Sorghum Rhizosphere Effects Reduced Soil Bacterial Diversity by Recruiting Specific Bacterial Species Under Low Nitrogen Stress.” Science of the Total Environment 770: 144742. 10.1016/j.scitotenv.2020.144742 33736399

[48] Fitzpatrick, Connor R. , Julia Copeland , Pauline W. Wang , David S. Guttman , Peter M. Kotanen , and Marc T. J. Johnson . 2018. "Assembly and Ecological Function of the Root Microbiome Across Angiosperm Plant Species." Proceedings of the National Academy of Sciences of the United States of America 115: E1157–65. 10.1073/pnas.1717617115 PMC5819437 29358405

[49] Hinsinger, Philippe , A. Glyn Bengough , Doris Vetterlein , and Iain M. Young . 2009. “Rhizosphere: Biophysics, Biogeochemistry and Ecological Relevance.” Plant and Soil 321: 117–52. 10.1007/s11104-008-9885-9

[50] Jiao, Shuo , and Yahai Lu . 2020. “Abundant Fungi Adapt to Broader Environmental Gradients Than Rare Fungi in Agricultural Fields.” Global Change Biology 26: 4506–20. 10.1111/gcb.15130 32324306

[51] Burns, Adam R. , W. Zac Stephens , Keaton Stagaman , Sandi Wong , John F. Rawls , Karen Guillemin , and Bohannan J. M. Brendan . 2016. “Contribution of Neutral Processes to the Assembly of Gut Microbial Communities in the Zebrafish Over Host Development.” The ISME Journal 10: 655–64. 10.1038/ismej.2015.142 26296066 PMC4817674

[52] Sloan, William T. , Mary Lunn , Stephen Woodcock , Ian M. Head , Sean Nee , and Thomas P. Curtis . 2006. “Quantifying the Roles of Immigration and Chance in Shaping Prokaryote Community Structure.” Environmental Microbiology 8: 732–40. 10.1111/j.1462-2920.2005.00956.x 16584484

[53] Putman, Lindsay I. , Mary C. Sabuda , William J. Brazelton , Michael D. Kubo , Tori M. Hoehler , Tom M. McCollom , Cardace Dawn , et al. 2021. “Microbial Communities in a Serpentinizing Aquifer are Assembled Through Strong Concurrent Dispersal Limitation and Selection.” mSystems 6: e00300–21. 10.1128/mSystems.00300-21 34519519 PMC8547479

[54] Neutel, Anje‐Margriet , Johan A. P. Heesterbeek , and Peter C. de Ruiter . 2002. “Stability in Real Food Webs: Weak Links in Long Loops.” Science 296: 1120–3. 10.1126/science.1068326 12004131

[55] Rooney, Neil , Gabriel Gellner Kevin McCann , and C. Moore John . 2006. “Structural Asymmetry and the Stability of Diverse Food Webs.” Nature 442: 265–9. 10.1038/nature04887 16855582

[56] Stouffer, Daniel B. , and Jordi Bascompte . 2011. "Compartmentalization Increases Food‐Web Persistence." Proceedings of the National Academy of Sciences of the United States of America 108: 3648–52. 10.1073/pnas.1014353108 PMC3048152 21307311

[57] Herren, Cristina M. , and D. McMahon Katherine . 2017. “Cohesion: A Method for Quantifying the Connectivity of Microbial Communities.” The ISME Journal 11: 2426–38. 10.1038/ismej.2017.91 28731477 PMC5649174

[58] Hernandez, Damian J , Aaron S. David , Eric S. Menges , Christopher A. Searcy , and Michelle E. Afkhami . 2021. “Environmental Stress Destabilizes Microbial Networks.” The ISME Journal 15: 1722–34. 10.1038/s41396-020-00882-x 33452480 PMC8163744

[59] Yuan, Mengting M. , Xue Guo , Linwei Wu , Ya Zhang , Naijia Xiao , Daliang Ning , Shi Zhou , et al. 2021. “Climate Warming Enhances Microbial Network Complexity and Stability.” Nature Climate Change 11: 343–8. 10.1038/s41558-021-00989-9

[60] Gang, Chengcheng , Wei Zhao , Ting Zhao , Yi Zhang , Xuerui Gao , and Zhongming Wen . 2018. “The Impacts of Land Conversion and Management Measures on the Grassland Net Primary Productivity Over the Loess Plateau, Northern China.” Science of the Total Environment 645: 827–36. 10.1016/j.scitotenv.2018.07.161 30031340

[61] Gao, Yang , Jimin Cheng , and Wei Liu . 2011. “Community Characteristics of Different Types of Grassland in the Loess Plateau.” Pratacultural Science 28: 1066–9. http://cykx.lzu.edu.cn//article/id/7a07b8b1‐2277‐4907‐80cf‐e65714b39d5e

[62] Zhong, Yangquanwei , Weiming Yan , Ruiwu Wang , Wen Wang , and Zhouping Shangguan . 2018. “Decreased Occurrence of Carbon Cycle Functions in Microbial Communities Along with Long‐Term Secondary Succession.” Soil Biology and Biochemistry 123: 207–17. 10.1016/j.soilbio.2018.05.017

[63] Nelson, Douglas W. , and L. E. Sommers . 1982. Total Carbon, Organic Carbon, and Organic Matter, Methods of Soil Analysis, Part 29, Chemical and Microbiological Properties, 539–79. Madison, WI: American Society of Agronomy and Soil Science Society of America. 10.2134/agronmonogr9.2.2ed.c29

[64] Bremner, John, M. , and C, S. Mulvaney . 1982. “Nitrogen‐Total.” In Methods of Soil Analysis, Part 31, Chemical and Microbiological Properties, edited by A.L. Page , 595–624. Madison, WI: American Society of Agronomy and Soil Science Society of America. 10.2134/agronmonogr9.2.2ed.c31

[65] Bolyen, Evan , Jai R. Rideout , Matthew R. Dillon , Nicholas A. Bokulich , Christian C. Abnet , Gabriel A. Al‐Ghalith , Alexander Harriet , et al. 2019. “Reproducible, Interactive, Scalable and Extensible Microbiome Data Science Using QIIME 2.” Nature Biotechnology 37: 852–7. 10.1038/s41587-019-0209-9 PMC7015180 31341288

[66] Jousset, Alexandre , Christina Bienhold , Antonis Chatzinotas , Laure Gallien , Angélique Gobet , Viola Kurm , Küsel Kirsten , et al. 2017. “Where Less May Be More: How the Rare Biosphere Pulls Ecosystems Strings.” The ISME Journal 11: 853–62. 10.1038/ismej.2016.174 28072420 PMC5364357

[67] Bokulich, Nicholas A. , Benjamin D. Kaehler , Jai Ram Rideout , Matthew Dillon , Evan Bolyen , Rob Knight , Gavin A. Huttley , et al. 2018. “Optimizing Taxonomic Classification of Marker‐Gene Amplicon Sequences with QIIME 2's q2‐Feature‐classifier Plugin.” Microbiome 6: 1–17. 10.1186/s40168-018-0470-z 29773078 PMC5956843

[68] Price, Morgan N. , S. Dehal Paramvir , and P. Arkin Adam . 2010. “FastTree 2—Approximately Maximum‐Likelihood Trees for Large Alignments.” PLoS One 5: e9490. 10.1371/journal.pone.0009490 20224823 PMC2835736

[69] R Core Team . 2018. “R: A Language and Environment for Statistical Computing.” R Foundation for Statistical Computing.

[70] Chen, Tong , and Luqi H. Yong‐Xin Liu . 2022. “ImageGP: An Easy‐to‐Use Data Visualization Web Server for Scientific Researchers.” iMeta 1: e5. 10.1002/imt2.5 PMC10989750 38867732

[71] Chen, Tong , Haiyan Zhang , Yu Liu , Yong‐Xin Liu , and Luqi Huang . 2021. "EVenn: Easy to Create Repeatable and Editable Venn Diagrams and Venn Networks Online.” Journal of Genetics and Genomics 48: 863–6. 10.1016/j.jgg.2021.07.007 34452851

[72] Oksanen, Jari , F. Guillaume Blanchet , Michael Friendly , Roeland Kindt , Pierre Legendre , Dan McGlinn , Peter R. Minchin , et al. 2020. “The Vegan Package”. Community Ecology Package. https://github.com/vegandevs/vegan

[73] Anderson, Marti J. , and J.Willis Trevor . 2003. “Canonical Analysis of Principal Coordinates: A Useful Method of Constrained Ordination for Ecology.” Ecology. 84: 511–25. 10.2307/3107905

[74] Breiman, Leo , Michael Last , and John Rice . 2003. “Random Forests: Finding Quasars.” In Statistical Challenges in Astronomy, edited by Eric D. Feigelson , G. Jogesh Babu . New York, NY: Springer. 10.1007/0-387-21529-8_16

[75] Dini‐Andreote, Francisco , James C. Stegen , Jan Dirk van Elsas , and Joana Falcão Salles . 2015. “Disentangling Mechanisms That Mediate the Balance Between Stochastic and Deterministic Processes in Microbial Succession.” Proceedings of the National Academy of Sciences of the United States of America 112: E1326–32. 10.1073/pnas.1414261112 25733885 PMC4371938

[76] Stegen, James C. , Xueju Lin , Jim K. Fredrickson , Xingyuan Chen , David W. Kennedy , Christopher J. Murray , Mark L. Rockhold , et al. 2013. “Quantifying Community Assembly Processes and Identifying Features That Impose Them.” The ISME Journal 7: 2069–79. 10.1038/ismej.2013.93 23739053 PMC3806266

[77] Knights, Dan , Justin Kuczynski , Emily S. Charlson , Jesse Zaneveld , Michael C. Mozer , Ronald G. Collman , Frederic D. Bushman , et al. 2011. “Bayesian Community‐Wide Culture‐Independent Microbial Source Tracking.” Nature Methods 8: 761–3. 10.1038/nmeth.1650 21765408 PMC3791591

[78] Newman, Mark E. J. , and Juyong Park . 2003. “Why Social Networks are Different from Other Types of Networks.” Physical Review E 68: 36122. 10.1103/physreve.68.036122 14524847

[79] Yuan, Jun , Jun Zhao , Tao Wen , Mengli Zhao , Rong Li , Pim Goossens , Qiwei Huang , et al. 2018. “Root Exudates Drive the Soil‐Borne Legacy of Aboveground Pathogen Infection.” Microbiome 6: 156. 10.1186/s40168-018-0537-x 30208962 PMC6136170

[80] Bastian, Mathieu , Sebastien Heymann , and Mathieu Jacomy . 2009. “Gephi: An Open Source Software for Exploring and Manipulating Networks.” Presented at the Third International AAAI Conference on Weblogs and Social Media. https://gephi.org/

