# Supplementary material for: Differential microbial assembly processes and co‐occurrence networks in the soil‐root continuum along an environmental gradient
Source: Imeta. 2022 Apr 5;1(2):e18. doi: 10.1002/imt2.18 (PMC10989781; doi:10.1002/imt2.18)

**Supplementary materials**

**Title:** Differential microbial assembly processes and co-occurrence networks in the soil-root continuum along an environmental gradient

**Running title:** Root microbiota along an environmental gradient

**Authors’ names:** Yangquanwei Zhong^1^, Patrick O. Sorensen^2^, Guangyu Zhu^3^, Xiaoyu Jia^4^, Jin Liu^4^, Zhouping Shangguan^4^, Ruiwu Wang^1^, Weiming Yan^4*^

**Author affiliations:**

1 School of Ecology and Environment, Northwestern Polytechnical University, Xi’an, 710072, P.R. China

2 Earth and Environmental Sciences, Lawrence Berkeley National Laboratory, Berkeley, CA, USA.

3 College of Environment and Ecology, Chongqing University, Chongqing, 400044, P.R. China

4 State Key Laboratory of Soil Erosion and Dryland Farming on the Loess Plateau, Northwest A&F University, Yangling, Shaanxi 712100, P.R. China

***Corresponding author:**

Weiming Yan, E-mail: yanweiming0110@nwsuaf.edu.cn

Address: Xinong Rd. 26, Institute of Soil and Water Conservation, Yangling, Shaanxi 712100, PR China

**Figure S1 ﻿**Environmental factors across the study sites. (A) Principal component analysis of environmental factors, (B) Important environmental factor changes with principal component axis 1. BGB, below ground biomass; AGB, aboveground biomass; RN, root nitrogen; ROC, root organic carbon; NO_3_^-^, nitrite; NH_4_^+^, ammonium; TP, total phosphorus; TN, total nitrogen; BD, bulk density; MAP, mean annual precipitation; and MAT, mean annual temperature.

**
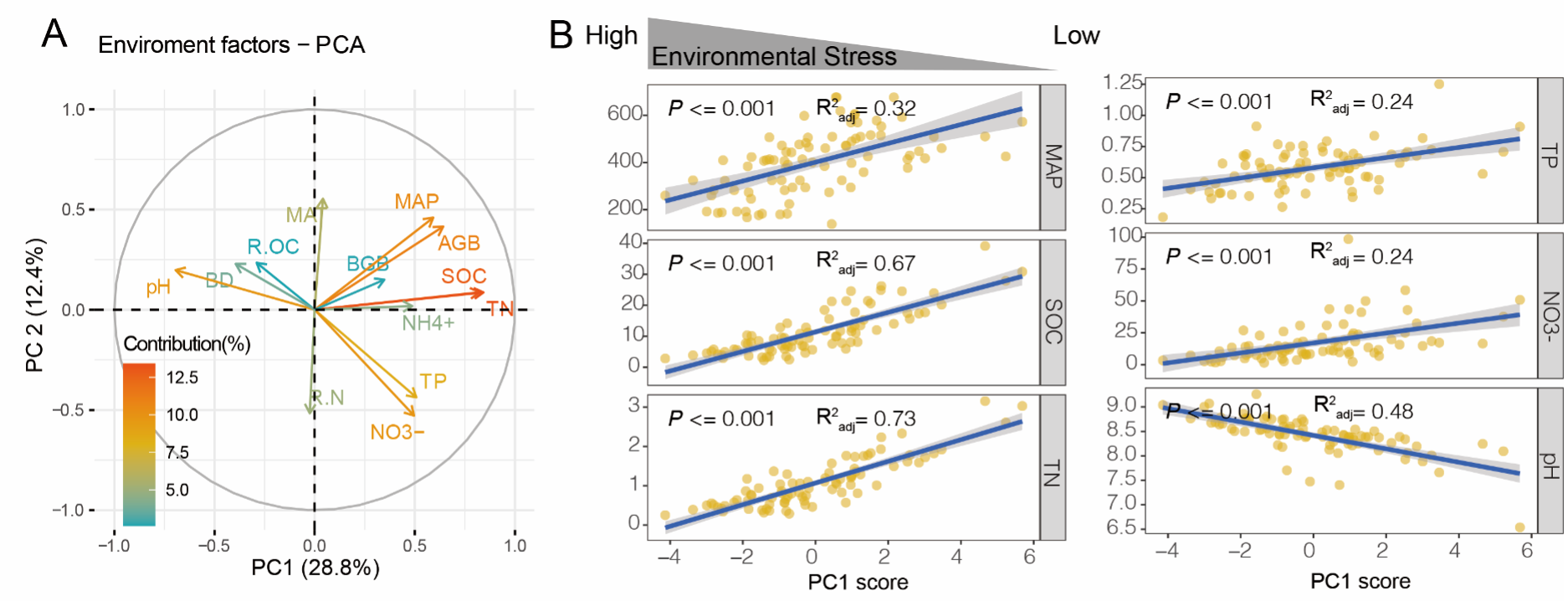
**

**Figure S2** Bacterial Shannon index among different microhabitats, grassland types and host plant types.


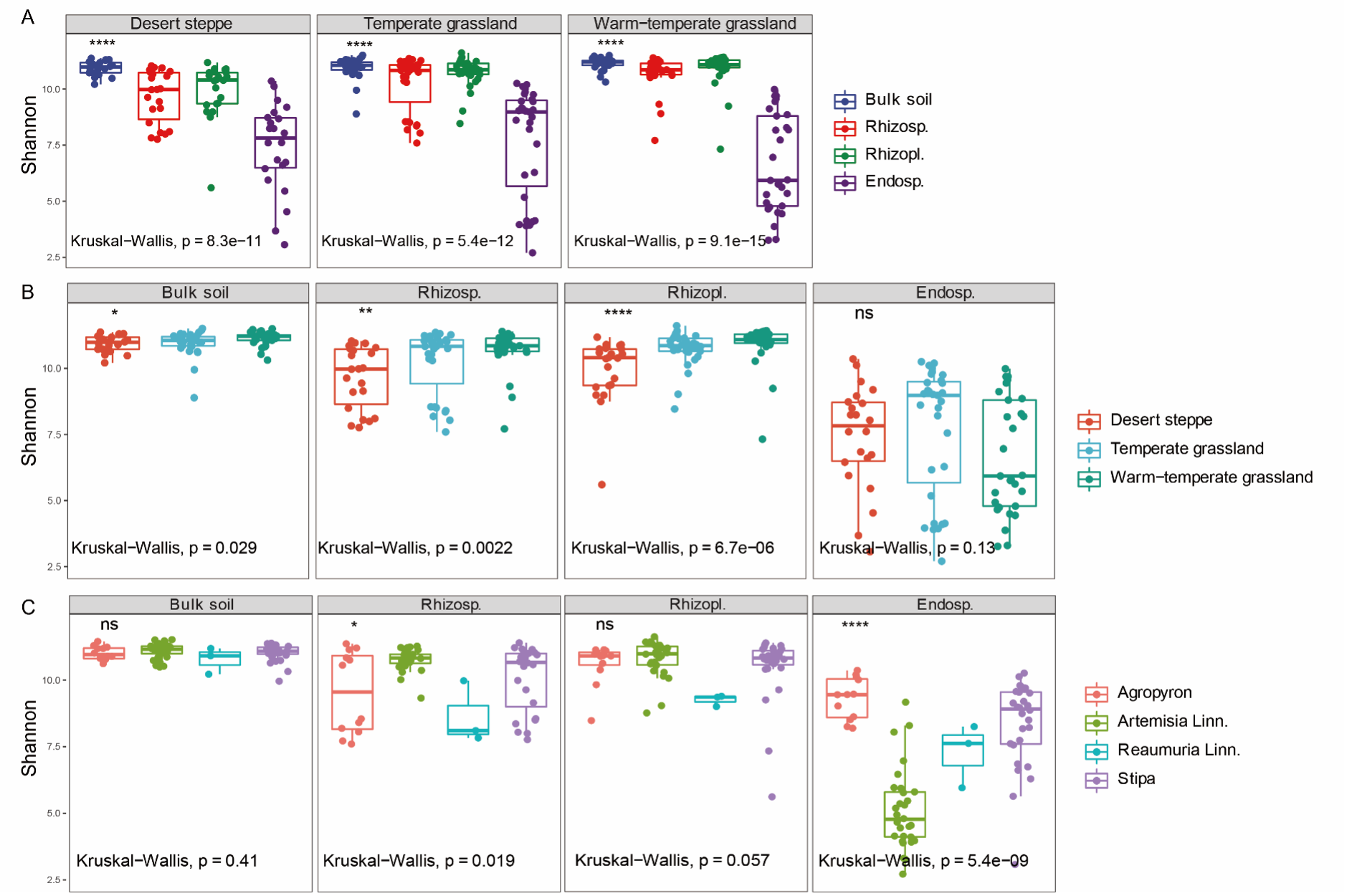


**Figure S3** Unconstrained PCoA with weighted UniFrac distances showing the soil and root microbiota community differences separated by microhabitat (A) and grassland type (B) and host plant (C). Ellipses cover 95% of the data for each group. Canonical analysis of principal coordinates (CAP) was used to better quantify the influence of these factors on the beta diversity.

**
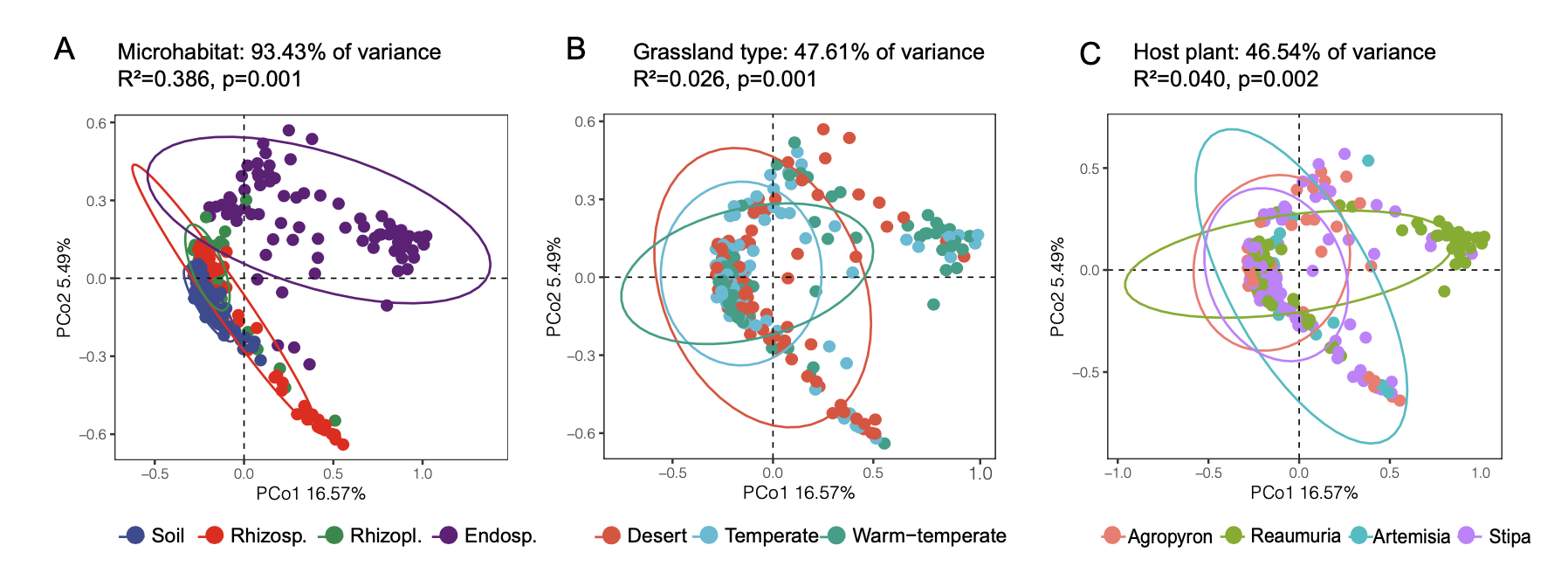
**

**Figure S4** Unconstrained PCoA with unweighted (A) and weighted (B) UniFrac distances showing the soil and root microbiota community differences separated by microhabitat (A), grassland type (B) and host plant (C). Ellipses cover 95% of the data for each group. Ellipses cover 95% of the data for each group.


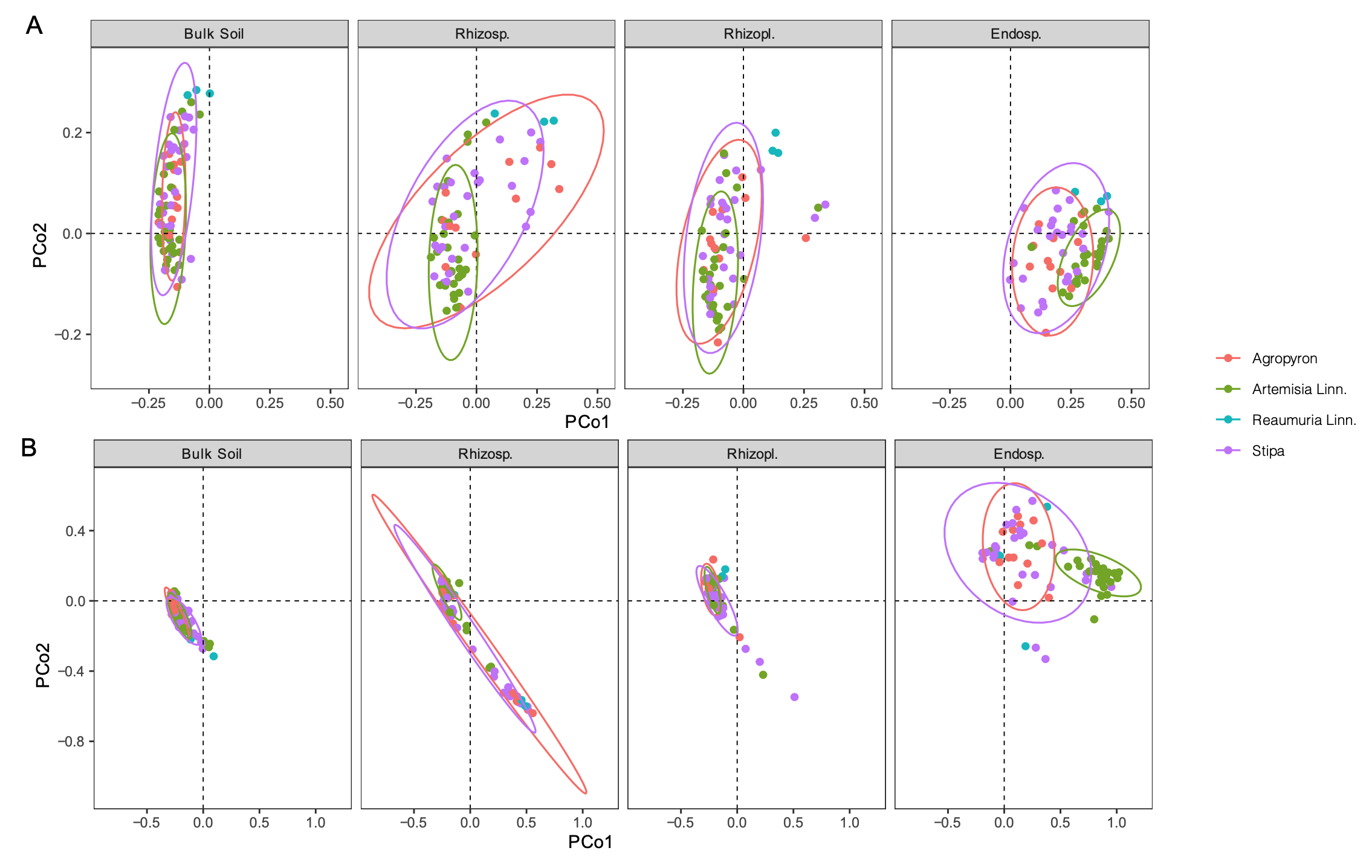


**Figure S5** The importance of environmental factors in predicting the relative abundance of bacterial community composition (X-axes (PC1) from the PCoA results) for the endosphere (A), rhizoplane (B), rhizosphere (C) and soil (D) communities. Non-significant (*p* > 0.05) relationships are shown as blank squares. BGB, belowground biomass, AGB, aboveground biomass, R. N, root nitrogen, R. OC, root organic carbon, NO_3_^-^, nitrite, NH_4_^+^, ammonium, TP, total phosphorus, TN, total nitrogen, BD, bulk density, MAP, mean annual precipitation, MAT, mean annual temperature.­


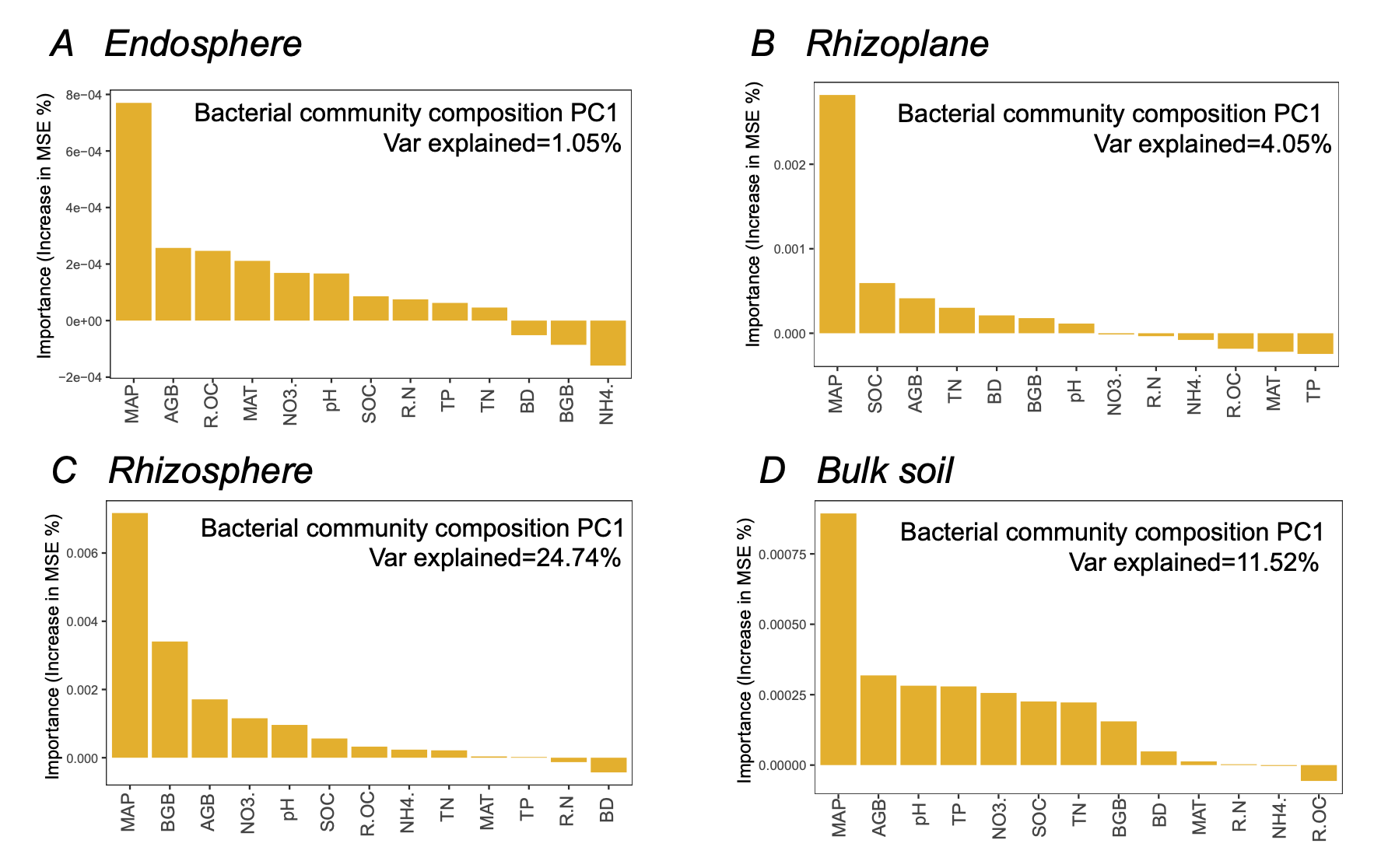


**Figure S6** Correlations among the Shannon index and environmental factors in each microhabitat based on the Spearman correlation coefficient. The color represents the value of the Spearman correlation coefficient; red indicates a positive correlation, and blue indicates a negative correlation. Non-significant (*p* > 0.05) relationships are shown as blank squares. BGB, belowground biomass; AGB, aboveground biomass; RN, root nitrogen; ROC, root organic carbon; NO_3_^-^, nitrite; NH_4_^+^, ammonium; TP, total phosphorus; TN, total nitrogen; BD, bulk density; MAP, mean annual precipitation; and MAT, mean annual temperature.


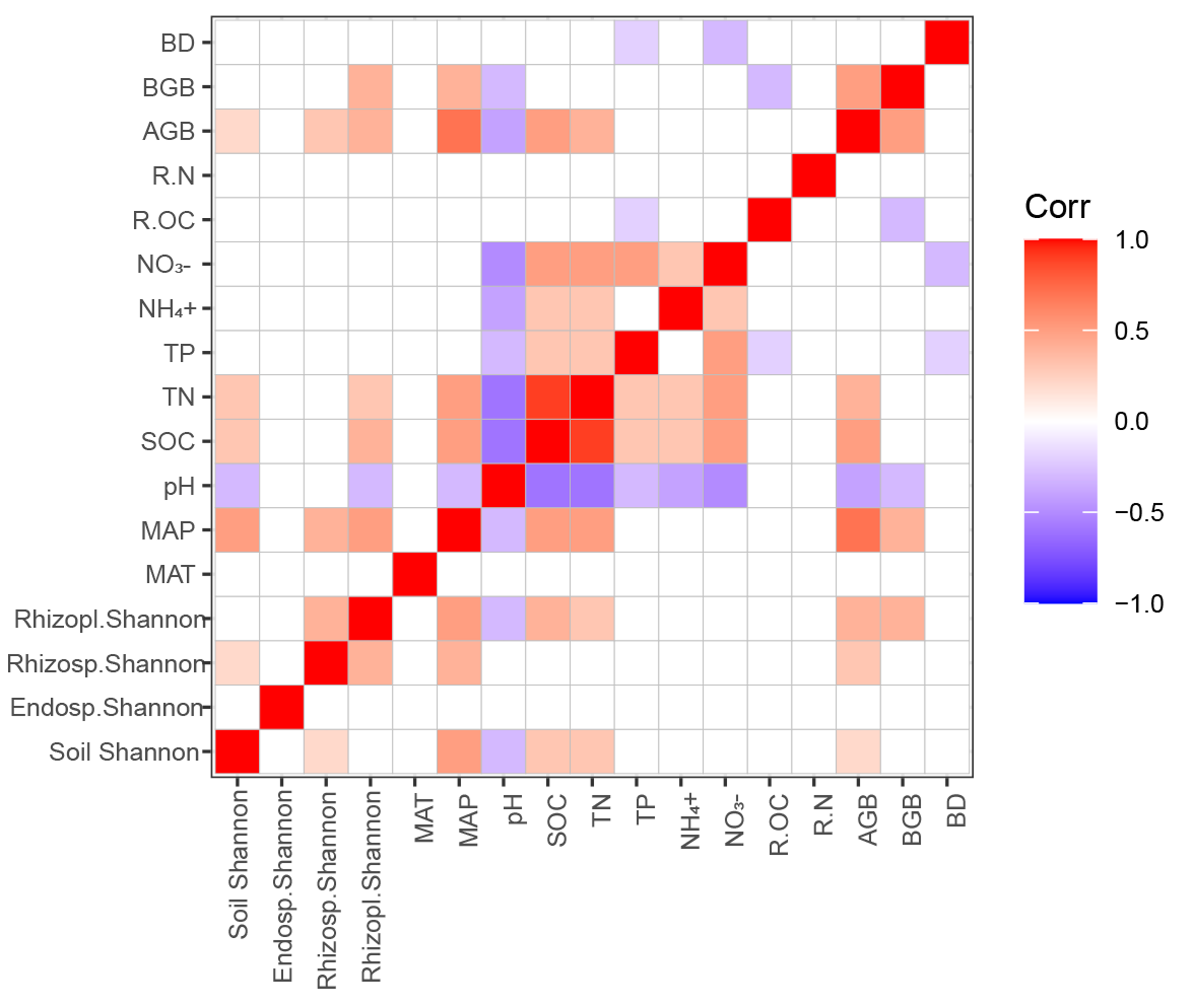

Supplement: Supplementary file 1 — Supplementary information. [file IMT2-1-e18-s002.docx]
